# Unusual Presentation of a Colonic Sarcoidosis

**DOI:** 10.1155/2012/169760

**Published:** 2012-03-26

**Authors:** Sami Daldoul, Wissem Triki, Kaouther El Jeri, Abdeljelil Zaouche

**Affiliations:** ^1^Department A of General Surgery, Charles-Nicole's Hospital, Boulevard 9-avril-1938, Tunis 1006, Tunisia; ^2^Tunis Faculty of Medicine, University of Tunis El-Manar, 15th Djebel Lakhdhar's Street, La Rabta, Tunis 1007, Tunisia; ^3^Department of Hepatology and Gastroenterology, Charles-Nicole's Hospital, Boulevard 9-avril-1938, Tunis 1006, Tunisia

## Abstract

Sarcoidosis is a multisystemic disorder of unknown cause that affects almost every tissue in the body. Colon is an extremely rare location of this disease. Clinical presentation, endoscopic appearances, and radiologic findings are not specific and may mimic much other affection. We report the case of a 64-year-old woman with inactive pulmonary sarcoidosis who presented alternating constipation and diarrhea. Colonoscopy revealed a stenotic tumor in the ascending colon. Histology failed to determine the nature of the lesion. Radiologic findings are those of a long stenotic tumor of the ascending colon associated with a multiple satellite lymphadenopathy. Endoscopic and radiologic descriptions are highly suggestive of a malignancy. The patient underwent a laparotomy, and a right hemicolectomy was performed. Examination of the resected specimen showed follicular structure with central epitheloid and giant cells and surrounding fibroblasts. These findings made the diagnosis of colonic sarcoidosis. The nonspecificity of the endoscopic and radiological signs of gastrointestinal sarcoidosis and the extreme rarity of colonic location make the preoperative diagnosis unlikely. The diagnosis will be then made only on histological examination of surgical specimens. We describe, through this observation, the results of paraclinical investigations that can suggest diagnosis and perhaps avoid surgery.

## 1. Introduction

Sarcoidosis is an inflammatory disease with unknown etiology. It is characterized by noncaseating granulomas in the absence of other autoimmune processes, infectious diseases, or foreign agents [1]. It primarily affects the lungs and lymphatic systems. The true incidence of intestinal involvement is not known, as the symptomatic intestinal disease is uncommon with only a few reported cases in literature. This incidence is estimated to less than 1%, and the colon is involved less frequently [1–3]. For this location, the preoperative diagnosis is difficult because of the nonspecificity of the clinical presentation, the nonspecific endoscopic findings, and, when histology failed, often appeared similarity with malignant lesions.

## 2. Case Report

A 64-year-old woman, with a 4-year case history of known pulmonary and cutaneous sarcoidosis, was admitted for abdominal pain, bloating and weight loss of nine-month duration.

The diagnosis of sarcoidosis was made based on the histological findings obtained from a transbronchial lung biopsy specimen. She was treated with Prednisone. She has been in remission since two years, and she was observed without medication. Pulmonary disease remained inactive.

The patient had developed diarrhea four months previously, which had seriously increased and often interrupted by episodes of obstinate constipation.

Physical examination revealed tenderness of the right lower quadrant with a mal-limited deep mass. Pelvic exam was normal. Pulmonary and cutaneous exam was normal.

**Figure 1:**
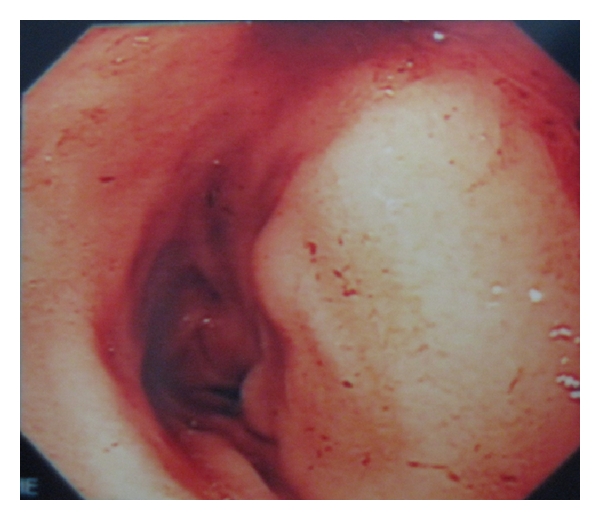
Colonoscopy findings showed a stenotic tumor in the ascending colon. The lesion was irregular and circumferential.

Total colonoscopy revealed a stenotic tumor in the ascending colon (Figure 1).

Biopsies from the tumor were taken, and histology of the specimens showed no evidence of neoplastic lesion, but an inflammatory infiltration of the submucosa which was rich of eosinophil cells.

**Figure 2:**
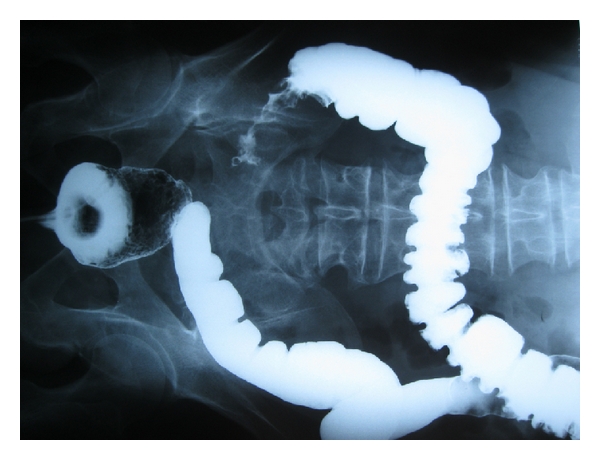
Contrast enema showed a long irregular stenotic lesion of the ascending colon.

Contrast enema showed a long irregular stenotic lesion of the ascending colon (Figure 2).

Abdominal ultrasound showed a 4 × 5 cm hypoechoic tumor in the ascending colon with no distant metastatic lesions.

**Figure 3:**
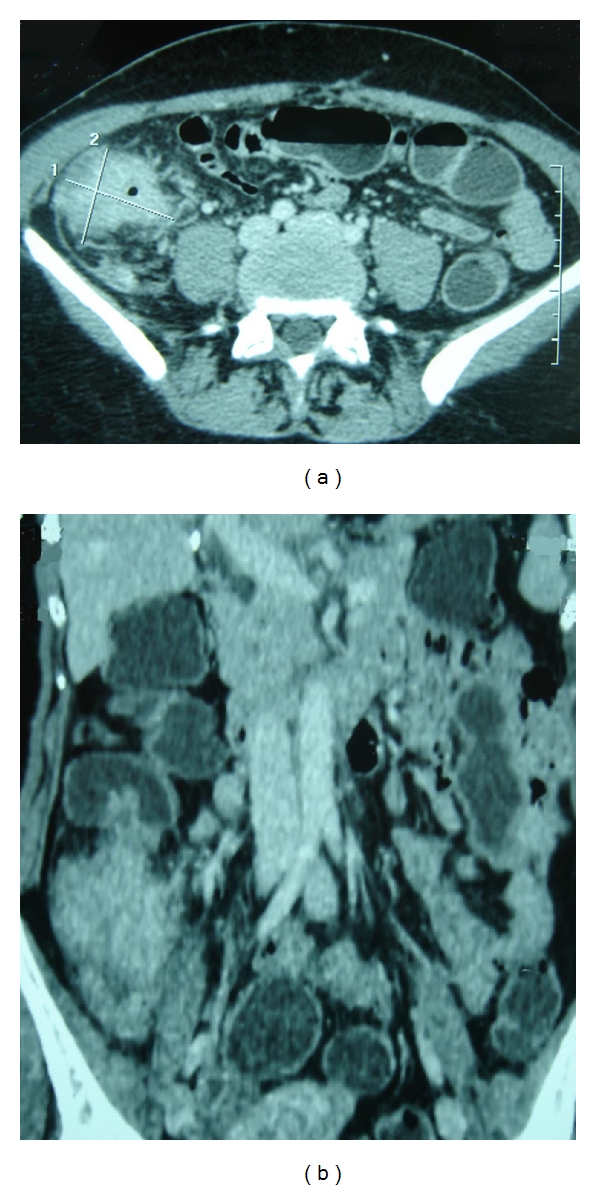
Transverse (a) and coronal, (b) CT images of the abdomen. Circumferential and irregular thickening of ascending colon miming a malignancy.

Abdominal computed tomography (CT) revealed a long stenotic tumor of the ascending colon extended 8 cm of length reducing notably the colonic lumen and associated with an infiltration of the adjacent fat tissues (Figure 3). There was a multiple satellite lymphadenopathy. CT showed no evidence of metastatic lesions.

The laboratory data revealed white cell count of 10200, hemoglobin 12.5 g/dL, normal liver tests, and a normal level of serum electrolytes. The carcinoembryonic antigen level was 3.6 ng/mL.

Relying on the endoscopic view and the scanographic appearance of the colonic lesion, and despite the short outcome of histology, the diagnosis of malignancy was made.

The patient underwent an exploratory laparotomy, which revealed constricting lesion of 8 cm in the midascending colon with numerous adjacent lymphadenopathy. A carcinologic right hemicolectomy was performed.

**Figure 4:**
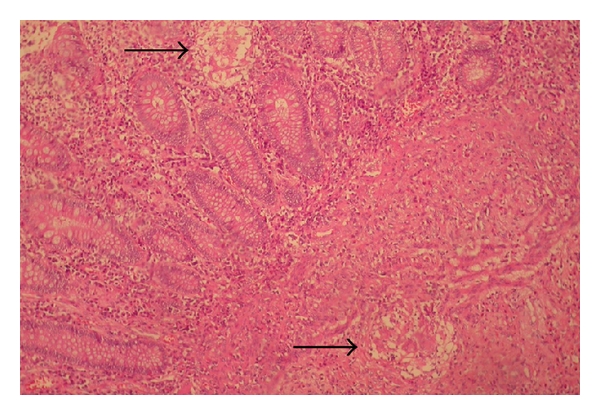
Noncaseating epithelioid granuloma (arrows) and multinucleated giant cells in the colonic submucosa with overlying normal colonic mucosa (hematoxylin and eosin, ×100).

Examination of the resected specimen revealed a thickening wall of the mildascending colon causing a long stenosis. The mucosa was dotted with a multiple superficial ulcerations. In microscopic exam, the submucosa showed follicular structure with central epitheloid, giant cells, and surrounding fibroblasts (Figure 4). There is no central caseation in the follicles. No acid-fast bacilli, or other organisms, or foreign bodies were identified. The appearances are those of sarcoidosis of the right colon.

 The subsequent evolution was favorable. The patient was discharged six days later.

## 3. Discussion

Sarcoidosis is a multisystemic disorder of unknown cause that is characterized by the formation of immune granulomas in involved organs.

Sarcoidosis may affect almost every tissue in the body, but it possesses a predilection for certain structures so that a disease pattern is presented. The lungs and the lymphatic system are the main affected sites [4–8].

Gastrointestinal tract is involved in less than 1% [1, 4, 6, 8, 9]. Involvement of the hollow organs is usually associated with concomitant pulmonary disease and is commonly asymptomatic. The stomach is the most commonly involved part of gastrointestinal tract although reported locations range from the esophagus to the rectum, sometimes diagnosed incidentally [1, 4, 7, 8]. Sarcoidosis of the colon is in fact rare and may mimic many other diseases.

For this location, literature is scanty. In a systemic revue of literature, Beniwal found 10 cases of colonic sarcoidosis from 1966 to 2003 [9]. The sigmoid colon is the site of most frequent involvement in the colon [1, 3, 9].

Clinical presentation is not specific, but the symptoms are those of a colonic disease. Indeed, patients were suffering from intermittent diarrhea, constipation, hematochezia, abdominal pain, distention, vomiting, and weight loss [1–3, 5–7, 10]. In our case, the lesion was symptomatic of alternating constipation and diarrhea.

Endoscopic appearances are variable but not suggestive of the diagnosis, and prior diagnosis of malignancy creates more diagnostic uncertainty.

It includes aphthous erosions or ulcers, friable mucosa or small punctuate bleeding sites miming colitis, plaque-like lesions, fold thickening, focal nodularity, or segmental narrowing, elevated lesions mimicking submucosal tumors [1–3, 7, 11]. The lesion can also resemble polyps [1, 11, 12] or may form irregular mass lesions mimicking carcinoma [8]. Strictures have resulted in obstruction mimicking carcinoma or a diverticular stricture [12]. It was the same for the treated case in which the stenotic lesion was highly suggestive of malignancy.

However, histological evidence of sarcoid granules has been found in apparently normal mucosa [2, 3, 12] so some authors speculate, for these cases, that colonic sarcoidosis first involved the submucosal lymph tissue and then will spread to a submucosal tumor [2].

In biopsy specimens, the presence of noncaseating granulomas containing multinucleated giant cells with little inflammation or crypt abscess formation is the pathologic hallmark of this disease [5, 7, 8]. The diagnosis is made if these histological findings are found in a patient with a compatible history. So, the pathologist must exclude the other granulomatous disorders such as tuberculosis, fungal infections, inflammatory bowel disease, malignancy, and delayed-type hypersensitivity to foreign antigens [1, 3, 5, 7, 9]. Crohn's disease and Wegener granulomatosis are rarely difficult to differ from sarcoidosis.

The utility of serum angiotensin-converting enzyme (SACE) level in differentiating sarcoidosis from Crohn's disease has been reported [9].

Serum ACE is thought to be produced by the epithelioid cells within sarcoid granulomas. It is elevated in approximately 60% of patients with sarcoidosis, and its level is thought to reflect whole-body granuloma mass and disease activity [7, 8].

Mentions of colonic sarcoidosis and corresponding radiologic descriptions are few. CT may show segmental or a symmetric wall thickening with preserved wall stratification [5]. These findings are not specific, and their differential diagnosis includes chronic inflammatory bowel disease, tuberculosis, and carcinoma. For our case, the tumor was unusual because it remains confined to the right colon wall with no extension to the adjacent structure despite its larger size up to 8 cm.

In case of appearance pseudotumor, if biopsy specimens fail to demonstrate the diagnosis of colonic sarcoidosis, surgical treatment with conventional surgical oncologic principle must be proposed for fear of carcinoma [10].

If not and if sarcoidosis is symptomatic, it can be treated with systemic corticosteroids [1, 4].

## 4. Conclusion

Colon is an uncommon site of gastrointestinal sarcoidosis. It should always be suspected for the tumor developing in colon in patients with the history of sarcoidosis especially when sarcoidosis lesions were found in several organs. The diagnosis is difficult to be made because of their high similarities in appearance with malignant tumor. The diagnosis should be suspected with compatible history patients if histology failed to show neoplastic proliferation. In these cases, only the presence of noncaseating granulomas will make the diagnosis. If not, patients should be treated as having a carcinoma.
